# A Method for Enhancing Inventory Efficiency of Densely Stacked Tags in RFID Cabinets

**DOI:** 10.3390/s25051617

**Published:** 2025-03-06

**Authors:** Chengzhen Ma, Jia Chai, Kaiqi Ren, Tingting Xie, Zhicheng Ruan, Yuzhu Liu, Dan Zhang, Suiping Jiang

**Affiliations:** 1 Beijing Institute of Computer Technology and Application, Beijing 100854, China; 2 Beijing Institute of Radio Metrology and Measurement, Beijing 100854, China

**Keywords:** inventory efficiency, radio frequency identification (RFID) cabinet, inventoried flags

## Abstract

This paper explicitly proposes a novel algorithm to enhance the inventory efficiency of densely stacked tags in a radio frequency identification (RFID) cabinet. By flexibly setting the inventoried flags, tags are not repeatedly inventoried by different interrogator antennas in the RFID cabinet. Comprehensive experiments are conducted to validate the proposed algorithm’s feasibility. The experimental results show that for 560 stacked tags, the proposed algorithm achieves 100% inventory accuracy while reducing inventory time by 40%, thereby significantly enhancing the efficiency of tag inventory management.

## 1. Introduction

The operating frequency of the high-frequency radio frequency identification (HF RFID) reader is set at 13.56 MHz. In the intricate scenario of dense stacking, tags are positioned within the alternating electromagnetic field generated by the reader’s antenna. They acquire energy through induced current and then smoothly conduct data interaction with the reader [1,2,3]. Within this specific frequency band, the magnetic field demonstrates an exceptional level of stability and a high degree of concentration within short-range regions. The signal is concentrated within a relatively small region, and the magnetic field interference between adjacent tags is relatively weak. This makes it particularly suitable for precisely reading single tags or a small number of tags in a densely stacked environment. In terms of environmental adaptability, the HF RFID system is extremely insensitive to liquids. In file storage areas, even when confronted with special conditions such as dampness, the normal reading of tags will not be affected at all, ensuring the stability and reliability of data collection. Regarding reading and writing performance, its reading range is highly controllable, effectively preventing misreading phenomena and enabling accurate identification of densely stacked RFID tags. In contrast, the ultra-high-frequency RFID reader operates based on the principle of electromagnetic wave radiation coupling. It features a relatively long signal wavelength and can transmit signals over long distances [4,5,6]. However, in a dense stacking scenario, electromagnetic waves are highly likely to experience reflection, scattering, and interference among tags, leading to signal chaos and significantly increasing the probability of misreading and missed reading. Therefore, in application scenarios that require penetration through multi-layer stacked environments, readers, with their unique advantages, demonstrate a broader application prospect.

HF RFID readers possess remarkable features including precise near-field identification, strong resistance to liquid and metal interference, and robust data security. Their superiority in reading densely stacked tags stands out prominently in file management scenarios [7]. In practical business operations, HF RFID reader antennas are typically installed at strategic locations within filing cabinets, such as the rear side. This ensures comprehensive coverage of all tag-bearing areas inside the cabinet. When it comes to file inventory or retrieval, the reader issues commands to the antenna, activating the densely stacked tags within its range. Leveraging their unique reading range and precision capabilities, HF RFID readers can accurately extract information from each tag and transmit these data to the backend management system [8,9]. Take a gigantic library as an instance. Prior to the adoption of HF RFID readers and the installation of antennas on bookshelves, manually inventorying and retrieving a vast collection of book files was not only time-consuming but also error-prone. After the implementation of these devices, the inventory of a single bookshelf can be completed within a few minutes. This has led to a substantial boost in both efficiency and accuracy. Moreover, when patrons search for books, the system, in tandem with the reader, can rapidly locate the desired items, significantly enhancing the library’s service quality. In the realm of intelligent warehousing, goods are predominantly stored in pallet-stacked configurations, each affixed with HF RFID tags. During the inbound process, the channel reader swiftly records the goods’ information. Warehouse personnel can then utilize the reader at any time to conduct inventory checks, thereby maintaining real-time awareness of inventory status. In the area of technological R&D, several foreign enterprises, led by NXP Semiconductors, have delved into research on near-field, highly dense stacked tags using HF RFID technology. The ILM series products they have developed are well equipped to meet the demands of highly dense stacking scenarios. Their reading accuracy and speed have reached a level suitable for initial commercial rollout. Regarding reading devices, low-power reader chips compliant with ISO international standards have been introduced. To date, the renowned semiconductor manufacturer NXP Semiconductors N.V. has delivered over one billion 13.56 MHz-operating RFID chips, namely ICODE chips, and this technology has been widely applied globally. Over 3000 libraries worldwide employ this technology for cataloging and identifying books and other media materials. In hospitals, it is used for patient identification, medical device tracking, and blood sample labeling. In factories, it enables the tracking and traceability of components during automated production processes.

In dense stacking scenarios, accuracy and stability are of utmost importance. In actual operations, to improve accuracy, inventory counts are often carried out multiple times, and the results are de-duplicated. Therefore, it is necessary to increase the inventory rate of readers in such scenarios. The inventory algorithm of readers is based on the load modulation method of sub-carriers [10,11] and adopts the traditional ALOHA tag inventory method. Currently, existing solutions mostly improve the inventory rate by optimizing the algorithm. For example, a distributed slotted-LBT (listen before talk) method has been proposed. This method aims to increase the number of readers that can read tags simultaneously. By setting up channel zones, it provides a stable channel-access time and reduces interference among readers. In addition, because each channel can be controlled separately, the classified channels can meet the different requirements of various applications [12]. There is also an improved dynamic frame slotted ALOHA algorithm for RFID. It can estimate the number of tags within the range of the reader, set the optimal frame length, enable the RFID system to achieve maximum throughput, and thus improve the tag identification efficiency [13]. Moreover, an anti-collision algorithm has been proposed, which combines the slot-based Bayesian estimation method with the improved frame slotted ALOHA. Compared with the Q algorithm currently used in the EPC global Gen-2 standard, this algorithm performs better in terms of system efficiency and time-based system efficiency [14]. In addition, an improved dynamic frame slotted ALOHA algorithm based on deep learning has been proposed. This system regards the number of tags in the reader’s range at different times as a time series and utilizes the advantages of a long short-term memory (LSTM) neural network in time series prediction to adjust the frame length [15].

However, existing solutions have limitations. Currently, readers communicate through a single antenna, first sending a Select command and then a BeginRound command to start the inventory [16,17,18,19]. This method is not suitable for scenarios where multiple reader antenna branches conduct polling for inventory. Take an RFID file cabinet as an example. It has multiple reader antenna branches, and every two reader antennas form a grid for placing tags. When a reader starts an inventory round, the tags placed in the same grid will be repeatedly inventoried by two adjacent reader antennas. The more times the tags are repeatedly inventoried, the lower the inventory efficiency. To solve the abovementioned problems, this paper proposes a method to change the tag inventory state by flexibly setting the inventory flags in the file cabinet. This method can effectively prevent tags from being repeatedly inventoried and improve the inventory efficiency of the RFID file cabinet.

## 2. Analysis

The RFID cabinet is divided into several grids by the interrogator antennas. Each tag is pasted on one paper with important information and stored in a grid in the cabinet. The interrogator can detect the presence of an individual tag or piece of paper by inventorying them.

**Figure 1:**
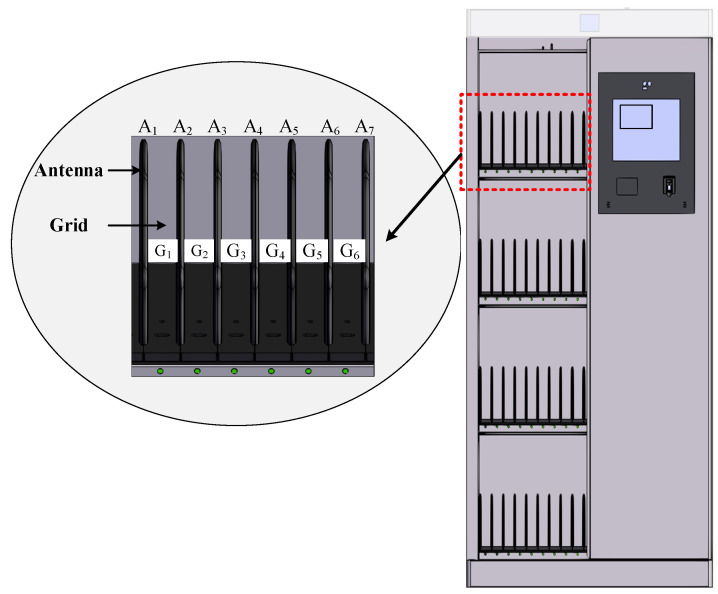
The RFID cabinet.

In practice, multiple interrogator antennas are deployed on each cabinet layer, and each layer is divided into several grids for placing the file paper. As shown in Figure 1, the cabinet has four layers, and seven interrogator antennas are deployed on the first layer, respectively represented by A_1_–A_7_. G_1_–G_6_ represent six separated grids. The interrogator communicates with the antenna A_1_–A_7_ successively to complete the inventory of all tags on the first layer of the cabinet.

According to the International Organization for Standardization (ISO) 18000-3 protocol, tags maintain an independent inventoried flag for each inventory session. Each of the two mandatory inventoried lags has two values, denoted as A and B. Sessions allow tags to associate a separate and independent inventoried flag to each of several interrogators. After successfully inventorying a tag, an interrogator may issue a command that causes the tag to set its inventoried flag for that session (A→B). Only tags with an inventoried flag of A respond in an inventory round [20,21,22,23].

**Figure 2:**
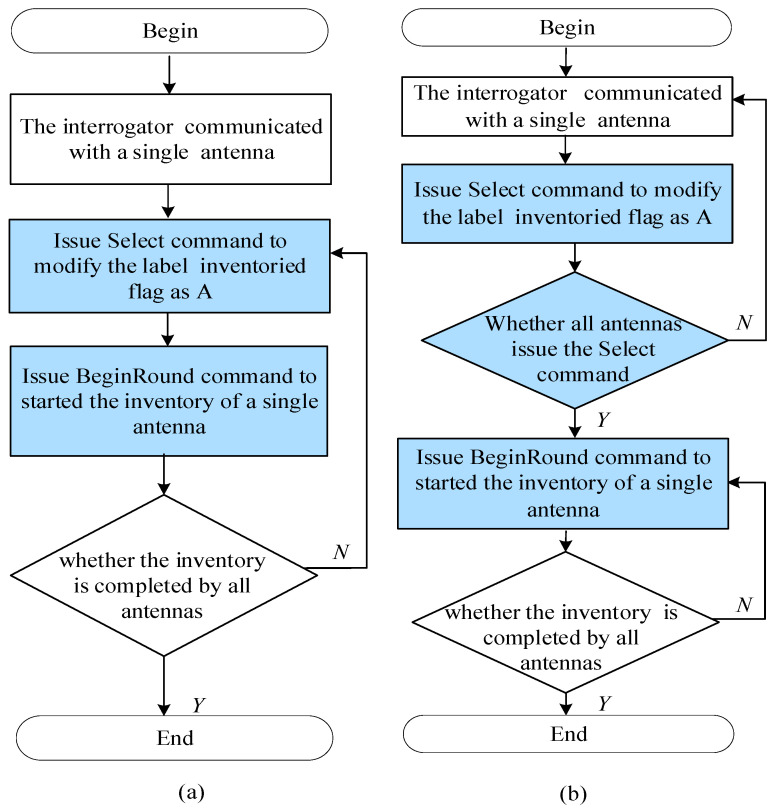
The algorithm (**a**) before optimization and (**b**) after optimization.

In the conventional algorithm, the interrogator issues commands to the antenna for the purpose of identifying the tags. Then, to take advantage of the unique electronic product code (EPC) of tags, duplicate tags are deleted in the host computer [24,25,26]. The interrogator issues the Select command through antenna A_1_ to set the inventoried flags of tags in grid G_1_ to A, and it then issues the BeginRound command to start a session. When the session ends, the inventoried flags of tags in grid G_1_ change from B to A. Then, the interrogator starts another session with antenna A_2_ to start the next round of inventory. However, the tags in grid G_1_ will also respond to the Select command of antenna A_2_, and the inventoried flags change from B to A, resulting in the tags in grid G_1_ being repeatedly inventoried by antennas A_1_ and A_2_. Similarly, the tags in grid S_i_ are repeatedly inventoried by antennas A_i_ and A_(i+1)_, where i is the antenna number, and i = 1, 2 … 6. Therefore, the tags in the cabinet are repeatedly inventoried by at least two different antennas, resulting in low inventory efficiency. The inventory algorithm is shown in Figure 2a.

To improve the inventory efficiency, an optimization algorithm is presented for tag inventory in an RFID cabinet. First, the interrogator communicates with the antennas A_1_–A_N_ in turn to issue only the Select command so that the inventoried flags of all tags on this floor of the cabinet are set to A. Then, the interrogator uses antenna A_1_ to issue the BeginRound command to start a round of inventory. After the end, the inventoried flag of each tag in grid G_1_ changes from A to B. Then, the interrogator issues the BeginRound command through antenna A_2_ to start the next round of inventory. The inventoried flag of each tag in grid G_1_ is B and cannot be inventoried again. Tags in grid G_2_ respond to the interrogator. This process is repeated until all antennas in the cabinet have completed the tag inventory procedure. The optimized algorithm is shown in Figure 2b.

In addition, the optimized algorithm has another advantage. Due to the limitation of the high-frequency interrogator’s transmitting power, a tag’s response signal far away from the interrogator antenna is weak, and it is easily submerged by noise, resulting in a decoding failure of the interrogator. According to the ISO 18000-3M3 protocol, the interrogator can only start the next round of inventory after a certain time delay after several failed decoding attempts [22,23]. This phenomenon will lead to a decline in inventory accuracy and low inventory efficiency because of multiple decoding attempts and delays of the interrogator, which is called the small-waveform problem. For the traditional algorithm, a single antenna simultaneously identifies the tags in the grid on its left and right sides. By contrast, for the optimized algorithm, a single antenna only identifies the tags in one side of the grid, and the tags with small-waveform problems are reduced; thus, the inventory efficiency is enhanced.

**Figure 3:**
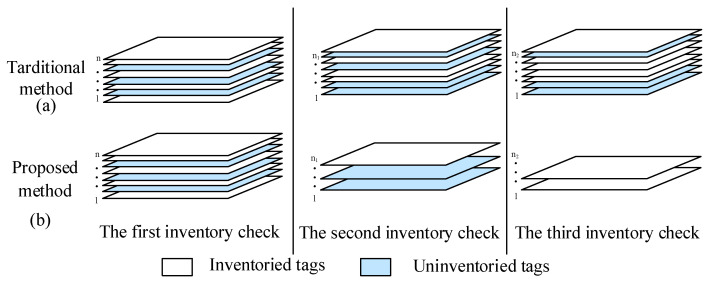
Schematic Diagram of Multi-tag Inventory.

In the tag inventory task, as the number of tags continues to increase, the proposed method is remarkable in improving the tag inventory efficiency compared with the traditional method. When the number of tags increases, a series of problems will be faced. Due to the limitations of the reader’s power and factors such as the frequency deviation of dense tags, it is easy to encounter missed readings during the tag inventory process, ultimately leading to inaccurate inventory results [27,28,29]. As shown as Figure 3a, in actual operation, for the traditional method, before each inventory, the reader first needs to set all the tags and then inventory all the tags within the inventory range. In order to improve the accuracy of inventorying, it is often necessary to conduct more than three consecutive repeated inventories. Finally, the results of the three inventories need to be de-duplicated in the host computer. Although this method indeed improves the accuracy of tag inventory to a certain extent, as the number of tags increases, its operation steps become cumbersome, resulting in extremely low inventory efficiency. As shown in Figure 3b, the operation of the proposed method is different. Before the first inventory, the reader also sets the tags and then inventories all the tags within the inventory range. However, during the second inventory, the tags are not set again, and only the tags that were not inventoried in the previous round are inventoried. In this way, the number of tags inventoried in each round is lower than that in the previous round, and the number of tags in the three rounds of inventory decreases successively. This operation mode effectively improves the accuracy and inventory rate of the reader.

## 3. Experimental Results and Discussion

### 3.1. Inventory Process

**Figure 4:**
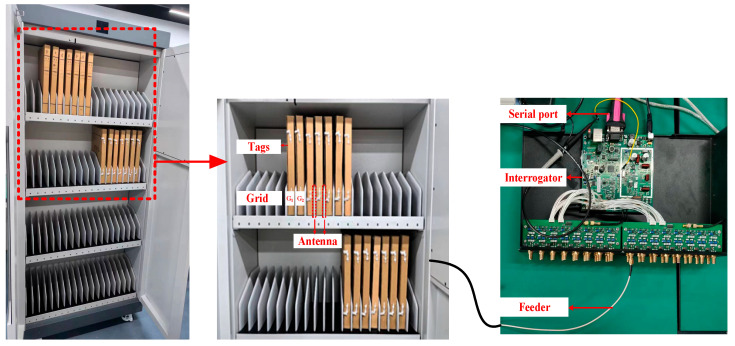
Experimental environment construction.

The method proposed in this paper is applied to a cabinet equipped with a high-power RFID interrogator, and the experimental environment is shown in Figure 4. The reader adopts a full digital signal processing design based on the software-defined radio architecture and is compatible with the ISO 18000-M3/EPC Class-1 HF protocol, boasting excellent performance. It performs outstandingly in radio frequency transceiver tasks and has the ability of low-loss switching for multi-channel antennas. The output power ranges from 5 W to 7 W, and it is equipped with 40 output channels, which are connected to an antenna board containing multiple antennas through 50 ohm feeder lines. This design enables it to be flexibly applied in different business scenarios and adapt to diverse product designs. In addition, the higher power of the reader can extend the tag setting time, providing a guarantee for the effective application of the proposed algorithm. The RFID tag is attached to the corner of the paper to identify the paper. A set of 80 pieces of paper is placed in a folder cable and stored in each grid of the cabinet. Multiple interrogator antennas are configured on each layer of the electronic cabinet. The interrogator is connected to the antenna via a feeder to provide energy and communication to the HF RFID tags. The computer system is connected to the interrogator.

A total of seven grids were tested, with 80 tags densely placed in each grid. When the accuracy and time of inventory identifying were tested, the average value of 15 inventory rounds was taken under the same conditions. The parameters of the interrogator are as shown in Table 1.

### 3.2. Simulation and Measurement Results

**Figure 5:**
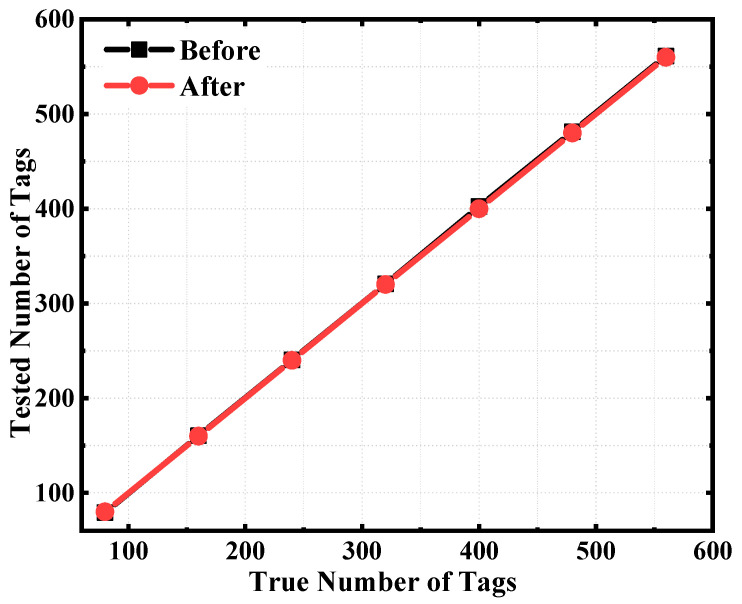
Comparison of inventory accuracy.

**Figure 6:**
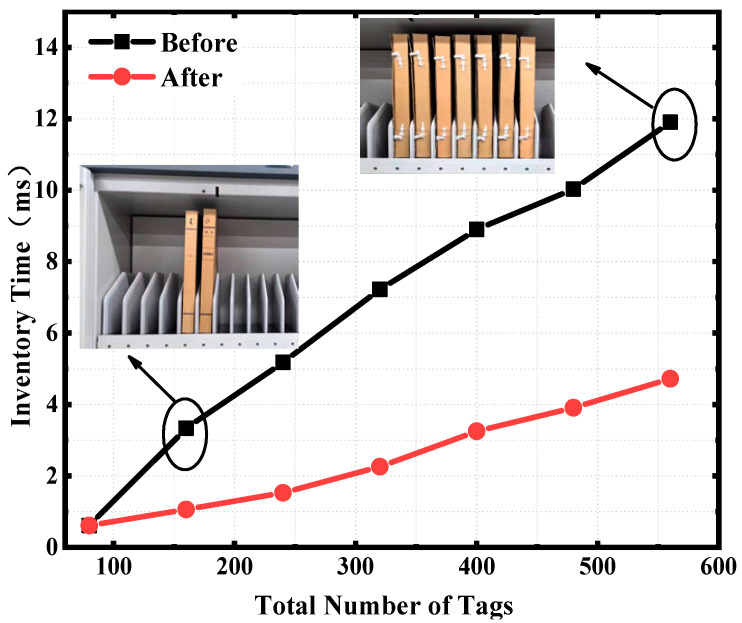
Comparison of inventory time.

As can be seen from Figure 5, the accuracy rate of the algorithm before and after optimization is 100% when there is only one grid, and to make the inventory results accurate, it is necessary to reassess the inventory results in the host computer to ensure the inventory accuracy before optimization. As can be seen from Figure 6, when there is only one grid for storing a total of 80 tags, the inventory times before and after enhancement are almost identical to 0.61 s. This is because there is only one grid to store tags, and there is no problem of duplicate inventories. When there are two grids to store a total of 160 tags, the inventory times before and after enhancement are 3.33 s and 1.06 s, respectively. The time optimization rate is 68%. When a total of 560 tags are stored in seven grids, the inventory times before and after enhancement are 11.9 s and 4.72 s, respectively. The time optimization rate is 60%. To enhance inventory efficiency in a short time, tags can be inventoried multiple times within the same time frame to improve accuracy. Completing more inventory tasks per unit of time can significantly boost the overall work efficiency. Take the management of museum cultural relic archives as an example. Saving a few seconds each time can lead to substantial savings in time costs in the long run. With the increase of the number of inventory antennas, the performance of the algorithm gradually increases.

**Figure 7:**
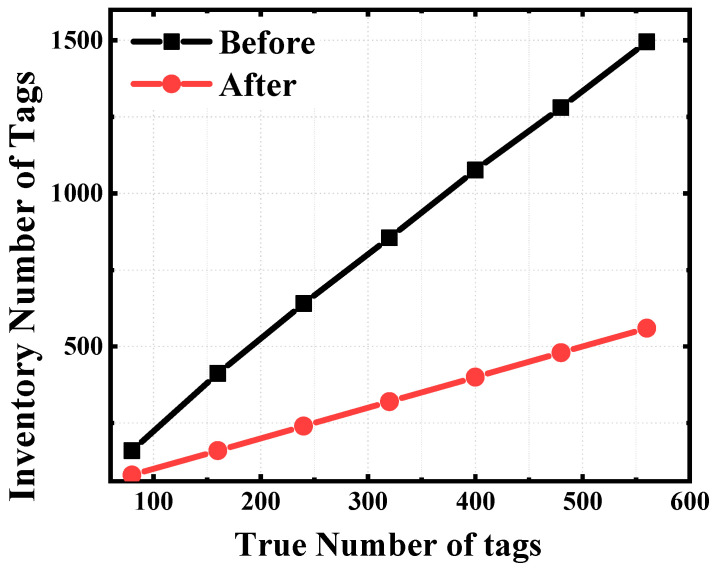
Comparison of the inventory results before removing duplicates.

As shown in Figure 7, for 560 tags, before optimization, the total inventory number is 1458, and the number of duplicate tags is 898. After optimization, the total inventory number is 560. Consequently, the quantity of tags subject to repeated inventorying is significantly diminished, leading to a notable enhancement of the overall inventory efficiency.

**Figure 8:**
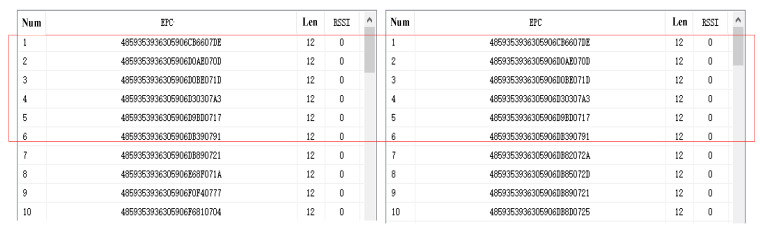
Duplicate EPC values of two different antennas in the upper computer.

The enhancement in inventory efficiency is mainly attributed to the optimization of repeated inventorying and addressing the small-waveform problem. In terms of the optimization of repeated inventorying, before the enhancement, the inventory range of antenna A_1_ is grid G_1_, and the inventory range of A_2_ includes grids G_1_ and G_2_, where the tags in grid G_1_ are repeatedly counted. As shown in Figure 8, the 10 EPC numbers in the inventory results of the two antennas were randomly intercepted. The six tags within the red box in Figure 8, with EPC numbers such as “4859353936305906CB6607DE” and “4859353936305906DOAEO7OD”, are repeatedly counted by two different antennas.

**Figure 9:**
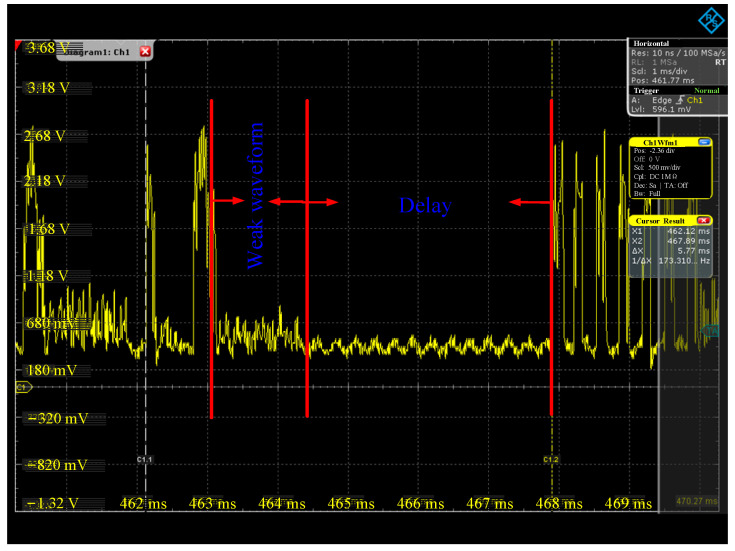
Schematic diagram of small-waveform problem displayed in oscilloscope.

In terms of small-waveform optimization, the waveform sampled from the oscilloscope is shown in Figure 9. The small waveform has lower amplitude and is submerged in noise, resulting in decoding failure by the interrogator, and then the delay is 3.58 ms. This is because, before optimization, there are tags that are far away from the interrogator antenna in grids G_1_ and G_2_ at the same time, and the tag response signal is weak. The interrogator fails to decode several times, and the next inventory is delayed for a period of time, resulting in a waste of time. After optimization, tags that have been inventoried do not respond repeatedly, and the number of small-wave tags is reduced, thereby enhancing the efficiency of tag inventory.

## 4. Conclusions

In order to solve the problem of low inventory efficiency caused by the repeated inventory of tags in multi-grid cabinets, a tag inventory method is proposed based on flexibly setting inventoried flag of each tag. The experimental results show that for 560 tags, the inventory time is shortened to 60% of the original under the condition of 100% inventory accuracy, which effectively enhances the efficiency of tag inventory. Therefore, the proposed method is applicable in RFID cabinets to manage densely stacked tags. The technology in this article starts with the common dense tag inventory model in document cabinets. It applies widely to almost all HF RFID scenarios such as file management, document verification desks, and medical supplies management, enabling fast inventory, information identification, and management.

## Figures and Tables

**Table 1:** Experimental parameters of the interrogator.

Name	Value
Air interface communications protocol	ISO 18000-3 Mode 3
Operating frequency	13.56 MHz
Reverse coding	Manchester coding
Output power	5–7 W
Modulation mode	Amplitude Shift Keying
Forward coding	Pulse coding
Reverse baud rate	106 kbit/s
Tari	10 μs

## Data Availability

Complete data are available in the research paper.
